# Changes in Thyroid Antibodies after Microwave Ablation of Thyroid Nodules

**DOI:** 10.1155/2022/7916327

**Published:** 2022-09-13

**Authors:** Zhen-Long Zhao, Ying Wei, Cai-Hong Liu, Li-Li Peng, Yan Li, Nai-Cong Lu, Jie Wu, Ming-An Yu

**Affiliations:** ^1^Department of Interventional Medicine, China-Japan Friendship Hospital, Beijing, China; ^2^Department of Ultrasound, Tumor Hospital of Mudanjiang City, Mudanjiang, Heilongjiang, China

## Abstract

**Purpose:**

Microwave ablation (MWA) is a minimally invasive method for the thermal ablation of benign thyroid nodules and papillary thyroid cancer (PTC) and has shown promising results. The aim of this study was to investigate the impact of MWA on thyroid antibodies and associated influencing factors.

**Materials and Methods:**

A total of 119 patients, including 69 with benign thyroid nodules and 50 with PTC, underwent MWA between June 2019 and June 2021. The serum levels of (free) triiodothyronine, (free) thyroxine, thyrotropin, and antibodies against Tg (TGAb), thyrotropin receptors (TRAb), and thyroid peroxidase (TPOAb) were measured during the follow up.

**Results:**

One month after ablation, three patients (4.3%) in the benign group had hypothyroidism, and one (1.4%) had hyperthyroidism. Four patients (5.8%) had subclinical hypothyroidism, and two (2.9%) had subclinical hyperthyroidism. Among the PTC patients, two (4%) had hypothyroidism, and one (2%) had hyperthyroidism. Two patients (4%) had subclinical hypothyroidism, and one (2%) had subclinical hyperthyroidism. In the benign group, among patients with normal preablation antibodies, the postablation TGAb abnormal rate was 12.7%, the TPOAb level was 4.8%, and the TRAb level was 0%. Among PTC patients, the postablation TGAb abnormal rate was 11.4%, the TPOAb level was 8.7%, and the TRAb level was 4.0%. The cutoff value of preablation TGAb for predicting postoperative antibody abnormalities was 19.0 IU/mL, while that of TPOAb was 11.4 IU/mL.

**Conclusions:**

MWA of thyroid nodules had little influence on thyroid function and antibodies. Elevations in TGAb, TPOAb, and TRAb beyond the normal ranges after MWA may be related to high preablation levels of TGAb and TPOAb.

## 1. Introduction

Thyroid nodules have shown a high incidence in recent decades. For symptomatic benign thyroid nodules and malignant thyroid nodules, hemithyroidectomy and thyroidectomy are commonly performed as standard and effective treatments [1–3]. However, hypothyroidism often develops after thyroidectomy and hemithyroidectomy [4]. In a large sample study, 34% of euthyroid patients developed hypothyroidism after hemithyroidectomy, 25% of whom needed levothyroxine [4].

Microwave ablation (MWA) has been widely used in the treatment of benign thyroid nodules [5] and papillary thyroid cancer (PTC) [6, 7]. Studies have demonstrated that MWA can preserve thyroid function in patients even with bilateral thyroid nodule ablation [8]. However, transient thyrotoxicosis has also been reported in a few cases [9]. Regarding thyroid antibodies, a small-sample study indicated that MWA of benign thyroid nodules may result in elevated thyroid antibodies, such as antibodies against thyroid peroxidase (TPOAb), thyroglobulin (TGAb), and thyrotropin receptor (TRAb) [10], while another study reported no significant change in TgAb and TPOAb [9]. To date, few studies have focused on thyroid function and antibody changes after MWA of thyroid nodules, especially PTC. In the present study, thyroid function and antibody changes following MWA were retrospectively analyzed, and possible factors influencing such changes were also investigated to identify predictors of thyroid function and thyroid antibody abnormalities after MWA.

## 2. Materials and Methods

This retrospective study was approved by the institutional review board of the hospital. Written informed consent was obtained from each patient before the procedure. The patients consented to the publication of their examination results and radiological images anonymously.

### 2.1. Patients

The clinical data of patients with thyroid nodules who underwent MWA between June 2019 and June 2021 were retrospectively reviewed. The inclusion criteria were as follows: (i) a diagnosis of benign nodules or PTC confirmed by US-guided fine-needle aspiration biopsy, (ii) refusal of or ineligibility for surgery, and (iii) a follow-up time of at least three months. The exclusion criteria were as follows: (i) a PTC tumor stage greater than T2N0M0, (ii) abnormal thyroid function before ablation, (iii) antithyroid drug or hormone replacement drug use before ablation, and (iv) incomplete follow-up data.

### 2.2. Preablation Assessment

A Logiq E9 ultrasound scanner (GE Healthcare, US) with a 9.0 MHz linear probe was used for the assessment. The three orthogonal diameters (the largest diameter and two perpendicular diameters) and the location of the nodules were measured and recorded. Each measurement was performed by three physicians, and the average was recorded as the final result.

Laboratory tests included complete thyroid function with triiodothyronine (T3), free triiodothyronine (fT3), thyroxine (T4), free thyroxine (fT4), and thyrotropin (TSH). Thyroid antibodies included TGAb, TPOAb, and TRAb. The normal ranges of the above parameters are listed in Table 1.

### 2.3. MWA Procedure

MWA was performed by two radiologists with more than 5 years of experience. Before ablation, contrast-enhanced ultrasound (CEUS) was performed to observe the enhancement mode and margin of the nodule. Thermal ablation was performed via MWA (Intelligent Basic Type Microwave Tumor Ablation System, Nanjing ECO Microwave System, Nanjing, China, or KY-2000 microwave system, Kangyou Medical, Nanjing, China). After ablation, CEUS was repeated to evaluate the ablation effect. Complete ablation was defined as a nonenhanced ablation zone completely covering the benign tumor and extending at least 2 mm from the original PTC margin on CEUS. At the end of the procedure, the puncture site was compressed for 30 minutes, and the patient remained under observation for 2 hours to monitor potential complications.

### 2.4. Postablation Assessment and Follow-Up Visits

At the clinical follow-up, various examinations, including ultrasound and thyroid function and thyroid antibody tests, were performed at 1, 3, 6, 9, and 12 months and every 6 months thereafter. Subclinical hyperthyroidism was diagnosed when TSH was lower than the normal range but (*f*) T3 and (*f*) T4 were normal, and subclinical hypothyroidism was diagnosed when TSH was higher than the normal range but (*f*) T3 and (*f*) T4 were normal.

### 2.5. Statistical Methods

Statistical analyses were performed using SPSS version 24.0 (IBM, Armonk, NY, USA). Normally distributed data are presented as the mean ± standard deviation (SD), and the median and (25–75% interquartile range (IQR)) were used if data did not follow a normal distribution. The independent two-sided Mann-Whitney *U* test and Wilcoxon signed-rank test were used to test differences between the medians of continuous variables for data that did not fit a normal distribution, and the two-sided Student's *t*-test was used for data following a normal distribution. The chi-square test and Fisher's exact test were used for categorical data. All differences were considered significant when *P* < 0.05.

## 3. Results

A total of 29 males and 90 females were enrolled in the study, including 69 patients with benign nodules and 50 with PTC. The median age was 44 years old (IQR 36–57, range 18–75). Complete ablation was achieved in all target tumors. The baseline characteristics of the enrolled patients in the benign and PTC groups are summarized in Table 2.

### 3.1. Thyroid Function

One month after ablation, three patients (4.3%, 3/69) in the benign group had hypothyroidism, and one patient (1.4%, 1/69) had hyperthyroidism. Two of these patients (one with hypothyroidism and one with hyperthyroidism) recovered without therapy at the 3-month and 6-month follow-ups. Hypothyroidism persisted in the remaining two patients at the end of the follow-up. In the PTC group, two patients (4%, 2/50) had hypothyroidism, and one patient (2%, 1/50) had hyperthyroidism. The patient with hyperthyroidism recovered without therapy at the 6-month follow-up. Hypothyroidism persisted in the remaining two patients at the end of the follow-up. The hyperthyroidism and hypothyroidism rates did not significantly differ between the benign and PTC groups after ablation.

One month after ablation, four patients (5.8%, 4/69) in the benign group had subclinical hypothyroidism, and two patients (2.9%, 2/69) had subclinical hyperthyroidism. Five of these patients (two with subclinical hyperthyroidisms and four with hypothyroidisms) recovered without therapy at the 3-month follow-up. At the end of the follow-up, only 1 patient still had subclinical hypothyroidism. In the PTC group, two patients (4%, 2/50) had subclinical hypothyroidism, and one patient (2%, 1/50) had subclinical hyperthyroidism. These patients all recovered without therapy. No significant difference was found regarding subclinical hyperthyroidism and hypothyroidism rates between the benign and PTC groups after ablation.

The incidence rates of thyroid function abnormalities after MWA of thyroid nodules are summarized in Table 3.

In the benign group, the baseline characteristics, preablation thyroid function, or preablation thyroid antibody tests did not significantly differ between the postablation euthyroid subgroup and the postablation (subclinical) abnormal thyroid function subgroup. In the PTC group, no significant differences were noted.

### 3.2. Thyroid Antibody Test

In the benign group, TGAb increased one month after ablation compared with the preablation value (preablation TGAb 10 (10–15.41) IU/mL vs. postablation TGAb 11.41 (10–18.22) IU/mL, *P* < 0.001). The one-month postablation TGAb was not significantly different between the normal postablation thyroid function group (postablation TGAb 11.41 (10–18.22) IU/mL) and the abnormal postablation thyroid function group (postablation TGAb 12.51 (10.30–110.26) IU/mL) (*P*=0.795). No significant difference was found between preablation and postablation TPOAb and TRAb values.

In the benign group, among patients with normal preablation antibodies, the postablation TGAb abnormality (out of reference range) rate was 12.7% (8/63), and 25% of the patients (2/8) recovered without treatment. The postablation TPOAb abnormality rate was 4.8% (3/63), and no patients had recovered at the end of the follow-up. No patient had postablation TRAb abnormalities.

In the benign group, no significant difference in baseline characteristics or preablation thyroid function was identified between the normal and abnormal postablation antibody groups.

After matching patients by age, maximum tumor diameter, ablation power, and ablation time with a 1 : 2 matching protocol without replacement between the abnormal postablation TGAb group and the normal TGAb group, the statistical results showed a significant difference in preablation TGAb and TPOAb values between the two groups. A similar significant difference was also found between the normal postablation TPOAb and abnormal TPOAb groups. The detailed results are shown in Table 4.

In the PTC group, TGAb increased one month after ablation compared with the preablation value (preablation TGAb 10 (10–14.40) IU/mL vs. postablation TGAb 11.81 (10–16.93) IU/mL), and the difference was significant (*P* < 0.001). The one-month postablation TGAb showed no significant difference between the normal postablation thyroid function group (postablation TGAb 11.77 (10–14.32) IU/mL) and the abnormal postablation thyroid function group (postablation TGAb 12.52 (8.66–32.13) IU/mL) (*P*=0.878). No significant difference was found between the preablation and one-month postablation TPOAb and TRAb values.

In the PTC group, among patients with normal preablation antibodies, the postablation TGAb abnormality rate was 11.4% (5/44), and 20% (1/5) of the patients recovered without treatment. The postablation TPOAb abnormality rate was 8.7% (4/46), and no patients had recovered at the end of the follow-up. The postablation TRAb abnormality rate was 4.0% (2/50), and 50% (1/2) of the patients had recovered at the end of the follow-up.

In the PTC group, the baseline characteristics or preablation thyroid function did not significantly differ between the normal postablation antibody group and the abnormal group.

After matching patients by age, maximum tumor diameter, ablation power, and ablation time with a 1 : 2 matching protocol without replacement between the abnormal postablation TGAb group and normal TGAb group, the statistical results showed a significant difference in preablation TGAb and TPOAb values between the two groups. Differences were also found in the preablation TGAb, TPOAb, and TRAb values between the normal and abnormal postablation TPOAb and TPOAb groups. The detailed results are shown in Table 5.

No significant differences in the incidence rates of postablation antibodies exceeding the normal ranges were observed between the benign nodule and PTC groups (*P* > 0.05).

**Figure 1:**
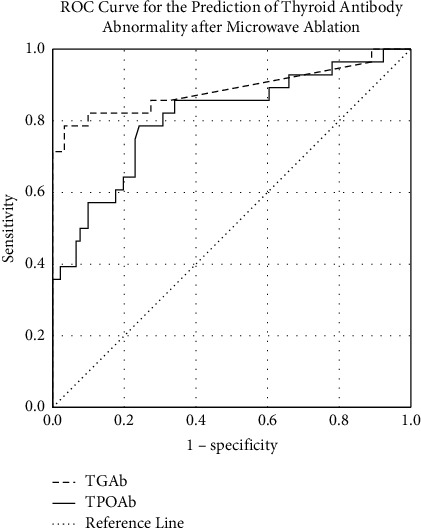
ROC curves for the prediction of postablation thyroid antibody abnormalities by thyroid peroxidase (TPOAb) and thyroglobulin (TGAb).

Among all 119 patients, 28 patients had abnormal postoperative antibodies (one or more abnormal antibodies among TGAb, TPOAb, and TRAb), and 91 patients had all normal values. Preoperative TGAb and TPOAb were used to predict postablation antibody abnormalities. ROC analysis was performed to evaluate the predictive efficacy. The cutoff value of TGAb was set to 19.0 IU/mL. The AUC was 0.887 (95% CI: 0.794–0.980), with a sensitivity of 0.786 and a specificity of 0.967. The cutoff value of TPOAb was set to 11.4 IU/mL. The AUC was 0.809 (95% CI: 0.709–0.909), with a sensitivity of 0.786 and a specificity of 0.758. The ROC curves are shown in Figure 1.

## 4. Discussion

MWA leads to tumor necrosis in situ. Coagulative necrosis of the thyroid microstructure occurs after ablation [11]. Follicles and follicular epithelial cells will lyse and be absorbed through the lymphatic system. Thyroid structure destruction may lead to exposure to thyroid antigens, such as thyroglobulin in follicles, as well as the cell membrane and intracellular antigens [10]. These antigens may stimulate or enhance local immune responses. In addition, MWA is a type of physical stimulation, and an inflammatory response band forms around the ablation zone, resulting in changes in local blood flow patterns and the aggregation of inflammatory cells and factors. The above changes may further stimulate or enhance the immune response to antigens. A recent study also demonstrated that MWA can result in the elevation of the neutrophil-to-lymphocyte ratio to affect the systemic inflammatory response and the ratio decreases along with tumor volume reduction [12].

The changes in thyroid function and antibody levels caused by ablation can be objectively demonstrated by laboratory examinations within one month after ablation. During the follow-up, some changes gradually recovered. The results of this study showed that MWA of thyroid nodules had little influence on thyroid function. The incidence of thyroid function abnormalities after MWA of benign and PTC is less than 6%. The functional changes were mild and did not require drug treatment. Most patients recovered during the follow-up. For benign or PTC nodules, various parameters, including sex, age, nodule size, ablation power, ablation time, preoperative thyroid function level, and preoperative antibody level, were not influencing factors of postoperative thyroid function abnormalities. Thyroid function abnormalities may be due to the influence of thermal stimulation on thyroid tissue, possibly resulting in an effect on thyroid blood supply patterns. For example, the original vessels that nourished target nodules may redistribute in the thyroid parenchyma, or the transient dysfunction of thyroid cells may occur around the ablation zone after thermal stimulation. However, most thyroid function abnormalities caused by these changes recovered along with the resolution of acute aseptic inflammation following heat stimulation, the recovery of cells from heat shock, and the adaptation of cells to blood supply redistribution.

The statistical results showed that in the benign and PTC groups, the value of TGAb increased slightly one month after ablation compared with that before ablation. However, no significant difference was noted in TPOAb and TRAb values, indicating that the release of thyroglobulin by MWA due to the destruction of the follicle structure had a slight impact on the immune response in thyroid tissue. However, the destruction of thyroid follicular epithelial cells, which release thyroid peroxidase and thyrotropin receptors, did not stimulate a marked immune response. Statistical analysis showed that the preablation TGAb and TPOAb levels in the abnormal postablation TGAb/TPOAb group were higher than those in the normal postablation TGAb/TPOAb group, and the difference was statistically significant. The above results indicate that the intensity of the local postablation immune response to thyroid thermal injury depends on the initial immune state in the preablation thyroid microenvironment. In other words, if the preablation TGAb and TPOAb levels were high, the antibody titer increased significantly after MWA. If the preablation antibody level was low, the postablation antibody was generally within the normal range. Further analysis showed that the risk of postoperative antibodies exceeding the reference ranges increased significantly when the preablation TGAb value exceeded 19.0 IU/mL or TPOAb exceeded 11.4 IU/mL.

The present study demonstrates that MWA can cause transient or persistent thyroid inflammation in some cases. Preexisting thyroid antibodies may lead to significant postablation thyroid antibody abnormalities. Therefore, a thyroid antibody test may be necessary before ablation, and patients should be informed of possible transient or persistent postablation thyroid antibody abnormalities even if preablation thyroid antibodies are within the reference range. Although rare, transient and persistent postablation thyroid function abnormalities also occurred in some cases, which may be another risk factor of MWA.

This study has several limitations. The retrospective study design may lead to selection bias. The reasons for postablation thyroid function and antibody abnormalities were mainly based on speculation instead of pathological and immunological evidence. Further basic studies are needed to investigate the fundamental cause of thyroid abnormalities after MWA.

## 5. Conclusions

MWA of benign and PTC nodules was safe and had little influence on thyroid function and thyroid antibodies. Most thyroid function abnormalities recovered during the follow-up. The elevation of TGAb, TPOAb, and TRAb beyond the reference ranges after MWA may be due to high preablation levels of TGAb and TPOAb.

## Figures and Tables

**Table 1:** Normal ranges of thyroid function parameters and thyroid antibodies.

Test item	Normal range
Triiodothyronine (T3)	(0.8–2.0) ng/mL
Free triiodothyronine (fT3)	(2.0–4.4) pg/mL
Thyroxine (T4)	(5.1–14.1) *μ*g/dL
Free thyroxine (fT4)	(0.93–1.7) ng/dL
Thyrotropin (TSH)	(0.27–4.20) *μ*IU/mL
Antibodies against thyroid peroxidase (TPOAb)	(0–34) IU/mL
Antibodies against thyroglobulin (TGAb)	(0–115) IU/mL
Antibodies against thyrotropin receptor (TRAb)	(0–1.75) IU/L

**Table 2:** Baseline characteristics of the enrolled patients.

Nodule type	Benign nodule (*n* = 69)	Papillary thyroid cancer (*n* = 50)
Age (years)	49.7 ± 14.0	40.2 ± 10.3
Gender (M : F)	16 : 53	13 : 37
Tumor location (*n*)		
Left lobe	26	16
Isthmus	2	2
Right lobe	41	32
Maximum diameter (cm)	3.1 (2.5–3.9)	0.7 (0.5–0.9)
Calcification	0 : 69	7 : 43
Enhancement mode		
Hypoenhancement	3	31
Isoenhancement	2	7
Hyperenhancement	64	12
Ablation time (s)	267.00 (159.50–467.50)	102 (54.5–140.5)
Ablation power (W)	50 (30–60)	30 (30–30)
Follow-up (month)	6 (3–6)	6 (3–9)

**Table 3:** Incidence rates of thyroid function abnormalities after microwave ablation of thyroid nodules.

Function abnormality	Benign (*n*)	Papillary thyroid cancer (*n*)	*P* (incidence rate between two groups)
Hyperthyroidism	1.4% (1/69)	2% (1/50)	0.623
Hypothyroidism	4.3% (3/69)	4% (2/50)	0.711
Subclinical hyperthyroidism	2.9% (2/69)	2% (1/50)	0.777
Subclinical hypothyroidism	5.8% (4/69)	4% (2/50)	0.986

**Table 4:** Preablation and postablation antibody values in different antibody groups after microwave ablation of benign nodules.

Antibody items	Value (IU/mL)	*P*
Preablation TGAb in the normal postablation TGAb group	10.00 (10.00–11.64) (*n* = 20)	<0.001
Preablation TGAb in the abnormal postablation TGAb group	83.44 (28.71–304.03) (*n* = 12)	

Preablation TPOAb in the normal postablation TGAb group	9.90 (6.43–18.26) (*n* = 20)	0.047
Preablation TPOAb in the abnormal postablation TGAb group	17.76 (10.65–77.44) (*n* = 12)	

Preablation TGAb in the normal postablation TPOAb group	10.00 (10.00–10.00) (*n* = 15)	0.001
Preablation TGAb in the abnormal postablation TPOAb group	77.94 (12.28–295.95) (*n* = 8)	

Preablation TPOAb in the normal postablation TPOAb group	5.00 (1.80–10.77) (*n* = 15)	0.001
Preablation TPOAb in the abnormal postablation TPOAb group	16.75 (6.54–251.43) (*n* = 8)	

**Table 5:** Preablation and postablation antibody values in different antibody groups after microwave ablation of papillary thyroid cancer.

Antibody items	Value	*P*
Preablation TGAb (IU/mL) in the normal postablation TGAb group	10.00 (10.00–12.03) (*n* = 17)	<0.001
Preablation TGAb (IU/mL) in the abnormal postablation TGAb group	193.90 (62.46–266.80) (*n* = 11)	

Preablation TPOAb (IU/mL) in the normal postablation TGAb group	9.64 (7.39–14.26) (*n* = 17)	0.019
Preablation TPOAb (IU/mL) in the abnormal postablation TGAb group	12.72 (11.66–135.20) (*n* = 11)	

Preablation TGAb (IU/mL) in the normal postablation TPOAb group	10.00 (10.00–11.64) (*n* = 15)	0.002
Preablation TGAb (IU/mL) in the abnormal postablation TPOAb group	150.10 (53.80–221.35) (*n* = 8)	

Preablation TPOAb (IU/mL) in the normal postablation TPOAb group	9.64 (5.86–14.05) (*n* = 15)	0.004
Preablation TPOAb (IU/mL) in the abnormal postablation TPOAb group	55.27 (12.54–197.53) (*n* = 8)	

Preablation TRAb (IU/L) in the normal postablation TPOAb group	0.30 (0.30–0.30) (*n* = 15)	0.016
Preablation TRAb (IU/L) in the abnormal postablation TPOAb group	0.46 (0.30–0.78) (*n* = 8)	

## Data Availability

The datasets used and/or analyzed during the current study are available from the corresponding author on reasonable request.
